# A Phase I Trial Assessing Lomecel-B Infusion in Individuals with Alzheimer’s Disease: Study Design and Rationale

**DOI:** 10.1093/geroni/igab046.2453

**Published:** 2021-12-17

**Authors:** Kevin Ramdas, Keyvan Yousefi, Ben Hitchinson, Lisa McClain-Moss, Liliana Diaz, Barry Baumel, Joshua Hare, Anthony Oliva

**Affiliations:** 1 Longeveron, Inc. , Miami, Florida, United States; 4 University of Miami, Miami, Florida, United States

## Abstract

Alzheimer’s disease (AD) is an irreversible neurodegenerative disorder characterized by memory loss and persistent cognitive dysfunction which significantly compromises quality of life. Brain inflammation is a prominent feature of AD pathology. Lomecel-B which is derived from culture-expanded medicinal signaling cells (MSCs) have immuno-modulatory capacity and control inflammation and the cytokine production of lymphocytes. The primary objective of this study was to evaluate the safety of Lomecel-B infused intravenously in individuals with AD. Safety was monitored by examining vital signs, physical and neurological exams, laboratory tests (hematology, coagulation, blood chemistry, and urinalysis). This was a multicenter phase 1 double-blinded, placebo controlled trial initiated with a safety run in phase of 3 individuals followed by a randomized phase of 28 individuals. During the safety run-in phase all subjects were treated with low dose Lomecel-B no less than 5 days apart, and evaluated for safety. In the randomized phase, subjects were treated with either low or high dose Lomecel- B or Placebo in a 2:2:1 randomization ratio. The study enrolled adults aged 50-80 years diagnosed with AD via confirmatory brain MRI and PET scan and a MMSE score of 18-24. Safety and efficacy assessments were completed at 30, 90, 180, 270 and 365 days. We describe the design and rationale for this phase 1 trial with the primary objective of assessing the safety of Lomecel-B on adults with AD. The secondary efficacy measurements included ADAS-Cog 11, MMSE, TMT, UPSIT, GDS, blood biomarkers and numerous quality of life questionnaires.

